# Detection of biological switches using the method of Gröebner bases

**DOI:** 10.1186/s12859-019-3155-0

**Published:** 2019-11-28

**Authors:** Yaman Arkun

**Affiliations:** Department of Chemical and Biological Engineering, Koc University, Rumeli Feneri Yolu, 34450 Sariyer, Istanbul Turkey

**Keywords:** Bistability, Output switchability, The Gröebner bases, Univariate basis polynomial, Steady-state solutions, Bifurcation, Polynomial equations, Biomolecular reactions

## Abstract

**Background:**

Bistability and ability to switch between two stable states is the hallmark of cellular responses. Cellular signaling pathways often contain bistable switches that regulate the transmission of the extracellular information to the nucleus where important biological functions are executed.

**Results:**

In this work we show how the method of Gröebner bases can be used to detect bistability and output switchability. The method of Gröebner bases can be seen as a multivariate, non-linear generalization of the Gaussian elimination for linear systems which conveniently seperates the variables and drastically simplifies the simultaneous solution of polynomial equations. A necessary condition for fixed-point state bistability is for the Gröbner basis to have three distinct solutions for the state. A sufficient condition is provided by the eigenvalues of the local Jacobians. We also introduce the concept of output switchability which is defined as the ability of an output of a bistable system to switch between two different stable steady-state values. It is shown that bistability does not necessarily guarantee switchability of every state variable of the system. We further show that, for a bistable system, the necessary conditions for output switchability can be derived using the Gröebner basis. The theoretical results are incorporated into an analysis procedure and applied to several systems including the AKT (Protein kinase B), RAS (Rat Sarcoma) and MAPK (Mitogen-activated protein kinase) signal transduction pathways. Results demonstrate that the Gröebner bases can be conveniently used to analyze biological switches by simultaneously detecting bistability and output switchability.

**Conclusion:**

The Gröebner bases provides a novel methodology to analyze bistability. Results clarify the distinction between bistability and output switchability which is lacking in the literature. We have shown that theoretically, it is possible to have an output subspace of an n-dimensional bistable system where certain variables cannot switch. It is possible to construct such systems as we have done with two reaction networks.

## Background

Bistable dynamical systems are frequently encountered in cellular processes. Information processing within cells is carried out by a complex network of switches and oscillators [1]. A bistable system is a system with two attractors. The system can switch between two distinct stable states without resting in intermediate states. Switch-like bistable responses have been observed in many applications including signal transduction [2–6], cell cycle control [7–13], learning and memory [14], growing bacterial biofilms [15], epileptic spike-wave discharges [16], neurons [17] and synaptic transmission [18]. Bistable switches have been designed synthetically as well. A genetic toggle switch in *Escherichia coli* has been constructed in [19]. The bistable switch forms an addressable cellular memory unit and has implications for biotechnology, biocomputing and gene therapy.

Considering its biological importance, significant research has been devoted to explaining the physical origin of bistability, to develop necessary conditions for its existence and to construct algorithms for its detection. In particular positive feedback and ultrasensitivity have been proposed as two necessary conditions for the physical appearance of bistability [20]. It is also well known that adding negative feedback to positive feedback can turn bistability into oscillations [21]. The theory of chemical reaction network (CRN) [22] proposes conditions for bistability by making use of the properties of a species-reaction graph. Angeli et al. [23] presented a graphical method to detect bistability for biological positive-feedback systems. Under some mild assumptions, if the open-loop response (when the positive feedback loop is opened) is monotone and has a sigmoidal shape, the system is guaranteed to be bistable for some values of the feedback gains. Finally, Wilhelm [24] proposed a smallest chemical reaction system with bistability. Two reactions constitute a positive feedback loop; a third reaction filters out small stimuli, and a fourth reaction prevents explosions. Analysis is based on the method of Instability Causing Structure Analysis (ICSA) which is based on the Jacobian of the reaction network. Recently a new method was proposed to study multistationarity and bistability of chemical reaction networks with few chemical complexes. The method uses polynomial systems with few distinct monomials and Gale duality [25].

The method of Gröebner bases was introduced by Buchberger in [26, 27] as a powerful computational tool to address fundamental questions in commutative algebra (polynomial ideal theory, algebraic geometry). Since its original inception, the method of Gröebner bases was applied to simplify the algorithmic solution of many difficult problems expressed in terms of multivariate polynomials. These include [28]: solving polynomial equations, coding theory, integer programming, partial differential equations, symbolic summation, graph theory and statistics. Today exploring its applicability to many diverse fields such as computational biology [29], chemical kinetics [30–32] and systems theory [33–35] is an active area of research.

The objective of this work is to explore the method of Gröebner bases to analyze the bistability and output switchability of biological signaling systems. The utility of the method is demonstrated using several examples from the cellular signaling systems literature. Next we give working definitions and examples of bistability and output switchability.

### Bistability

Consider the dynamical system *S*_*f*_ expressed as DAE (Differential Algebraic Equations):

1 $$ \dot{x}=h\left(x,y\right)\kern3em x\in {R}^n $$

2 $$ 0=y+g(x)\kern0.5em y\in {R}^m $$

where Eq. (1) represents the dynamic mass balances with the nonnegative concentrations of species *x* and *y*. Eq. (2) is a set of algebraic constraints due to the species conservation laws. Substituting *y* from Eq. (2) into Eq. (1), one gets:

3 $$ \dot{x}=f(x)\ x\in {R}^n $$

The steady-states of *S*_*f*_ are the solutions of 0 = *f*(*x*).

A bistable system is a system with two attractors. In general, a plethora of interesting possibilities with different attractors and separatrices can result in different types of bistabilities which coexist in the parameter space of interest. Some of the popular bistabilities are between two stable fixed points [10, 11, 14], between a stable fixed point and a limit-cycle oscillator [15–17]; or between two stable periodic orbits [36, 37]. In this paper, we adopt the following definition for bistability:

**Definition 1:** The dynamical system *S*_*f*_ is *state bistable* if it has three nonnegative distinct real steady-states (*ss*) for the state *x,* two of which are stable (${x}_{ss}^{1,S}\ \mathrm{and}\ {x}_{ss}^{3,S}\Big)$ and one is unstable ${x}_{ss}^{2,U}$ where the superscript *S* and *U* denote stable and unstable, respectively.

We refer to such bistability as fixed-point bistability to distinguish it from other types of bistabilities mentioned above. Each stable steady-state (or fixed-point) has its own basin of attraction (i.e. the set of initial conditions that asymptotically converge to that steady-state). These basins are separated with a boundary defined by a separatrix. Most often, the separatrix contains a steady-state that is an unstable saddle-point [11]. Upon perturbations in the medium reflected by the parameter changes in the model, other types of attractors like limit cycles can be born from these fixed points via Hopf bifurcation [36]. For example, it was shown in [38] that bistability is a necessary condition for the emergence of oscillations in the MAPK cascade signaling.

Throughout the paper bistability will mean fixed-point state bistability.

### A one-dimensional example

**Fig. 1:**
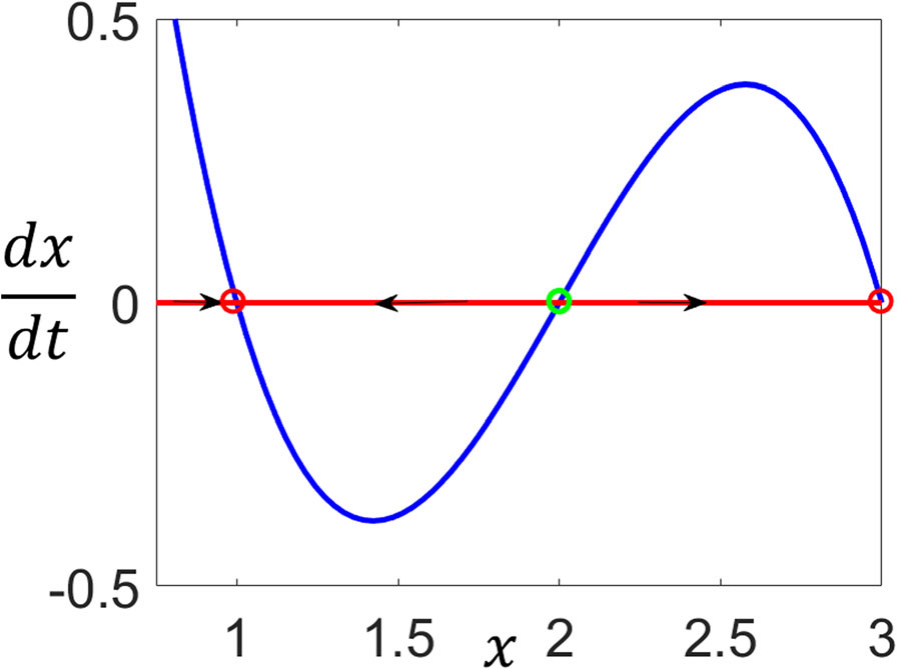
Bistability of a one-dimensional system. Two stable fixed points (red circles) separated by an unstable saddle-point (green circle)

Figure 1 shows a bistable system resulting from a one-dimensional ordinary differential equation $\frac{dx}{dt}=-{x}^3+6{x}^2-11x+6.$ There are three positive fixed points at *x* = 1, 2 and 3 . The unstable fixed point at *x*= 2 separates the basins of attraction of the stable fixed points. The trajectories starting from the initial conditions to the left of 2 approach the stable fixed point *x* = 1, and the trajectories starting from the initial conditions to the right of 2 approach the stable fixed point at *x* = 3.

### A two-dimensional example

In [24] a smallest chemical reaction system with bistability was proposed. The model consists of the following two-component mass-action kinetic ODE system:

4 $$ \frac{dx}{dt}=16y-{x}^2- xy-1.5x $$

5 $$ \frac{dy}{dt}={x}^2-8y $$

**Fig. 2:**
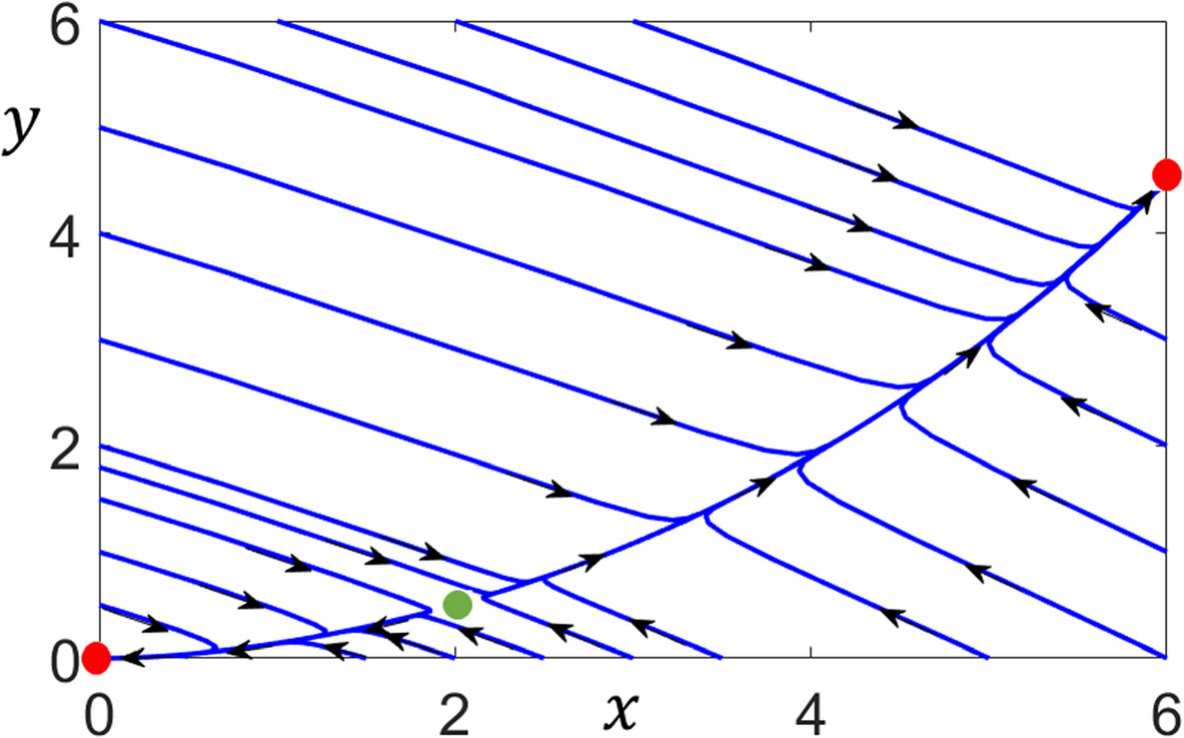
Phase-plane of a two-dimensional bistable system. The system has two stable fixed points (red circles) and an unstable saddle-point (green circle). Trajectories are separated into two basins of attraction of the stable steady-states

The system has three steady-states, two of which are stable at (0, 0), (6, 4.5) and an unstable steady-state which is a saddle-point at (2, 0.5). Figure 2 shows the phase plane with trajectories emanating from different initial conditions. Due to the saddle-point, the phase plane is divided into two basins of attraction which contain the trajectories approaching the two stable steady-states.

Bistability exits in models with higher dimensions *n* > 2 as well. For example, in [11] existence of a saddle point and two stable fixed points are highlighted with an apoptosis model that consists of 8 states. The authors present a local analysis to identify the saddle point that helps to understand the global properties of biological switches.

### Output switchability

Many biological events are binary with certain variables switching on and off between active and inactive states to perform important biological functions. For a bistable dynamical system *S*_*f*_, one is often interested in the switching response of the concentration of some species. Therefore, we include an output variable *y* in the model and the analysis. In general output *y* is taken to be any of the system states *x*_*i*_ *i* = 1 : *m*. Without loss of generality, the concentration of the first species, or the first state variable *x*_1_, is defined as the output. The new dynamical system with output *x*_1_ is denoted by ${S}_{f,{x}_1}$ and expressed as:

$$ \dot{x}=f(x)\ x={\left[{x}_1{x}_2\dots ..{x}_n\right]}^T $$

$$ y={x}_1 $$

Next, we introduce the following definition:

**Definition 2:** A bistable dynamical system ${S}_{f,{x}_1}$ is called *output switchable* if the steady-state output values *x*_1, *ss*_ are different at the two stable steady-states of the state *x* i.e. at (${x}_{ss}^{1,S}\  and\ {x}_{ss}^{3,S}\Big)$.

Differentiation between state bistability (Definition 1) and output switchability (Definition 2) is not made in the literature. For a one-dimensional system with a single output, bistability implies output switchability. However, for higher dimensional systems, bistability does not necessarily guarantee switchability for every output variable or state variable *x*_*i*_ *i* = 1 : *m*. Theoretically, it is possible to have an output subspace containing certain variables that do not switch.

## Results

The method of Gröebner bases (see Methods section) is applied to several systems to detect biological switches.

- *Bistable systems with switchable outputs.*

**Example 1.** In [24] a smallest bistable system is given that consists of the following four reactions:

$$ S+Y\overset{k_1}{\to }2X. $$

$$ 2X\overset{k_2}{\to }X+Y $$

$$ X+Y\overset{k_3}{\to }Y+P $$

$$ X\overset{k_4}{\to }P $$

The system is described by a two-component mass-action ODE system:

6 $$ \dot{x}={f}_1\left(x,y\right)=2{k}_1y-{k}_2{x}^2-{k}_3 xy-{k}_4x $$

7 $$ \dot{y}={f}_2\left(x,y\right)={k}_2{x}^2-{k}_1y $$

with *k*_1_ = 8, *k*_2_ = *k*_3_ = 1, *k*_4_ = 1.5.

Since the steady-states are determined by the solutions *f*_1_(*x*, *y*) = 0 and *f*_2_(*x*, *y*) = 0, the Gröbner basis is computed for these two polynomials using the reduced Gröebner basis program *gbasis* available in the Symbolic Math Toolbox of MATLAB:

8 $$ {g}_1\;(x)=8{x}^2-{x}^3-12x $$

9 $$ {g}_2\left(x,y\right)={x}^2-8y $$

Solving this triangular system and checking the eigenvalues of the Jacobians confirms that the system is bistable with three steady states:

$$ \left({x}_{ss}^{1,S},{x}_{ss}^{2,U},{x}_{ss}^{3,S}\right)=\left(\begin{array}{c}0\\ {}0\end{array}\right),\left(\begin{array}{c}2\\ {}0.5\end{array}\right),\left(\begin{array}{c}6\\ {}4.5\end{array}\right) $$

**Fig. 3:**
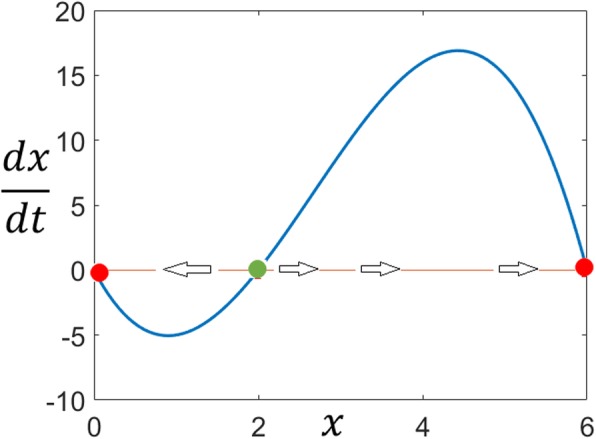
The univariate basis polynomial for Example 1. Two stable fixed points (red circles) separated by an unstable saddle-point (green circle)

Considering *x* as the output, the system is output switchable since *g*_1_(*x*) satisfies the necessary and sufficient conditions (Eqs. 59–61) for switchable outputs given in the Methods section. Plot of *g*_1_(*x*) with its three distinct roots is given in Fig. 3.

Note that the inverse problem of given a “desirable” univariate basis polynomial such as (8), reconstruction of a corresponding reaction network is possible although this network is not unique in general. The cubic depletion term −*x*^3^ suggests a bilinear term (−*xy*) where *y* is proportional to *x*^2^ so that (−*xy*) =  − *x*^3^ . This can be realized by the following set of reactions:

$$ S+Y\overset{k_1}{\to }2X $$

$$ 2X\overset{k_2}{\to }X+Y $$

$$ X+Y\overset{k_3}{\to }Y+P $$

Steady-state mass balance for *y* gives $y=\left(\frac{k_2}{k_1}\right){x}^2=\frac{x^2}{8}.$ The third reaction provides the cubic depletion rate $-{k}_3 xy=-{k}_3\left(\frac{k_2}{k_1}\right){x}^3=-\frac{x^3}{8}$ *.* Without this reaction the system can not have three steady-states and bistability is not possible. The first two reactions also provide the quadratic production term *x*^2^ for the species *x.* Finally, a first order reaction*:*

$$ X\overset{k_4}{\to }P $$

gives the linear depletion rate −1.5*x.* Summing up all the terms yields

*g*_1_(*x*) = *x*^2^− $\frac{x^3}{8}-1.5x$ which has the same solutions as the targeted univariate basis (8). Note that in [24] the above smallest bistable system is constructed in a similar way but without introducing the Gröebner basis.

In order to check if the output *y* is switchable, we change the lexicographic monomial order of the unknown variables (*x*, *y*) and recompute the Gröebner basis where the univariate polynomial is now a function of *y*:

10 $$ {g}_1(y)=-2.25y+5.{y}^2-1.{y}^3 $$

11 $$ {g}_2\left(x,y\right)=x-4.33y+0.66{y}^2 $$

Since *g*_1_(*y*) satisfies the conditions for switchable outputs (Eqs. (59–61)), the system is switchable in the output *y* as well.

**Fig. 4:**
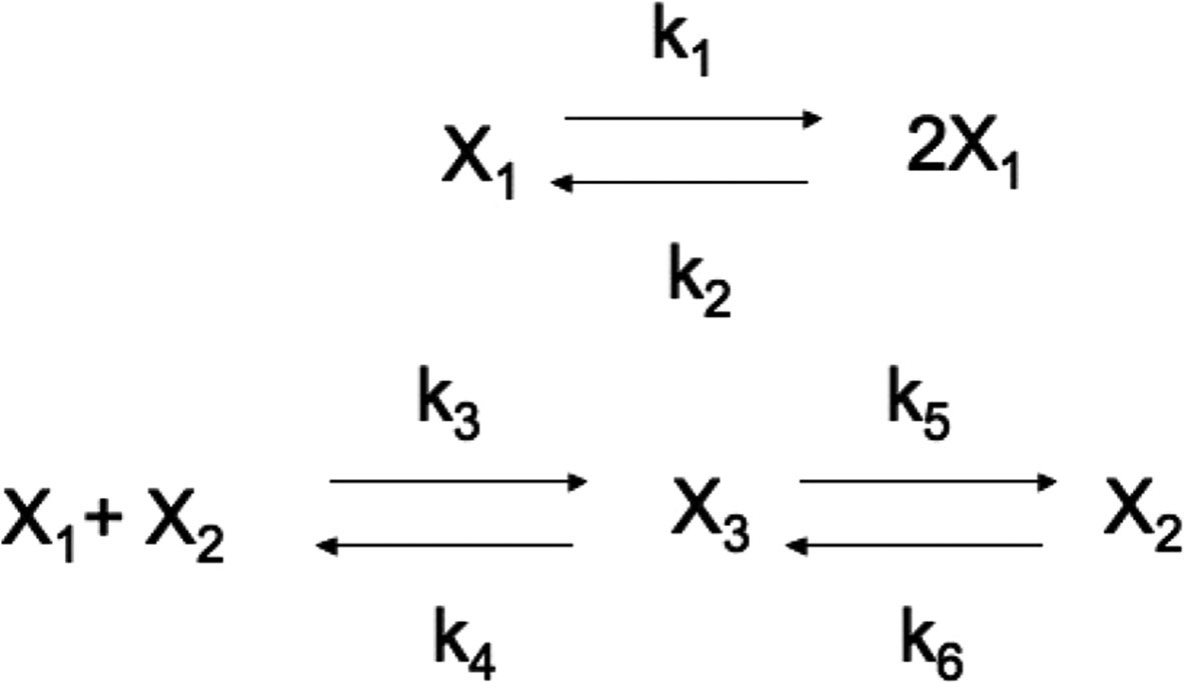
Edelstein Chemical Reaction Network [27]. Parameters: *k*_1_ = 8.5, *k*_2_ = *k*_3_ = *k*_4_ = *k*_5_ = 1, *k*_6_ = 0.2

**Example 2**. Consider the Edelstein reaction scheme analyzed in [29] and given in Fig. 4.

The system is described by the following DAEs:

12 $$ \dot{x_1}={k}_1{x}_1-{k}_2{x}_1^2-{k}_1{x}_1{x}_2+{k}_4{x}_3 $$

13 $$ \dot{x_2}={k}_4{x}_3+{k}_5{x}_3-{k}_3{x}_1{x}_2-{k}_6{x}_2 $$

14 $$ {x}_2+{x}_3=c=30. $$

Substituting the parameter values (see Fig. 4) and eliminating *x*_3_, one gets:

15 $$ \dot{\ {x}_1}=8.5{x}_1-{x}_1^2-{x}_1{x}_2-{x}_2+30 $$

16 $$ \dot{x_2}=-{x}_1{x}_2+60-2.2{x}_2 $$

The Gröbner basis is calculated as

17 $$ {g}_1\left({x}_1\right)=6.3{x}_1^2+6-11.3{x}_1-{x}_1^3 $$

18 $$ {g}_2\left({x}_1,{x}_2\right)=-{x}_2+0.833{x}_1^2-7.0883{x}_1+25 $$

The system is bistable with three steady states for the state *x*:

$$ \left({x}_{ss}^{1,S},{x}_{ss}^{2,U},{x}_{ss}^{3,S}\right)=\left(\begin{array}{c}1\\ {}18.75\end{array}\right),\left(\begin{array}{c}1.63\\ {}15.62\end{array}\right),\left(\begin{array}{c}3.66\\ {}10.23\end{array}\right) $$

**Fig. 5:**
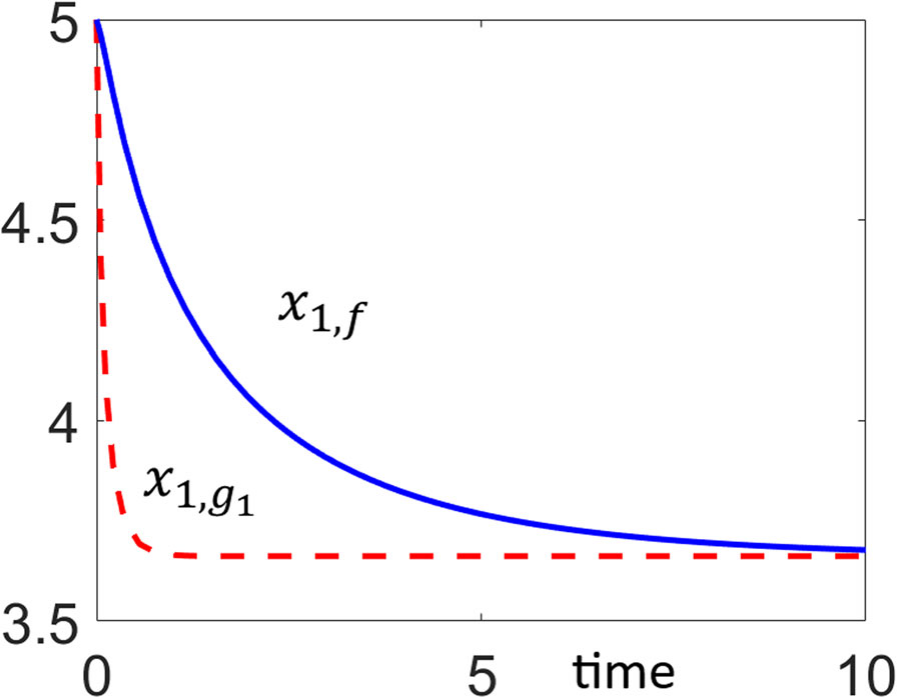
Comparison of the output responses of the original system (*x*_1, *f*_) and the univariate dynamical system (${x}_{1,{g}_1}$). Responses start from an initial condition and converge to one of the stable fixed points

In the Methods section we derive that for a bistable system to have a switchable output, the rate of generation of the output must consist of a quadratic and a constant term, and the rate of depletion must consist of a cubic and a linear term. Considering *x*_1_ as the output in the current example, the system is output switchable since *g*_1_(*x*_1_) has quadratic and constant generation, and linear plus cubic depletion terms (see (17)), and it satisfies the necessary and sufficient conditions (Eqs. 59–61). The dynamic output responses of the original system (*x*_1, *f*_) and the univariate basis polynomial dynamical system (${x}_{1,{g}_1}$) are compared in Fig. 5 for one initial condition. Trajectories converge to the same stable steady-state.

b. *Bistable systems with unswitchable outputs.*

It is difficult to find physical examples in the literature for bistable systems with unswitchable output(s). There are several reasons for this seemingly lacking data. First, only the switching variables are analyzed to show bistability; therefore, even if there are some outputs (or states) that do not switch, they are not reported. Second, it is possible that some outputs lose their switchability under abnormal conditions only (e.g. disease states due to mutations etc.) that create the right conditions for the emergence of unswitchable output(s). As a result, no distinction is made in the literature between bistability and output switchability. However, as we have presented above, the two concepts are not the same. In addition, at a practical level, for a complex system with many states, it is plausible for some output(s) of a bistable sytem to keep the same steady-state values as the system state switches from one stable steady-state to another. These special outputs can be acting as chaperons that change their values in transient only in order to help the other outputs to switch, and when they complete their tasks they return to their steady-states.

**Example 3**. The smallest bistable system whose output is not switchable is given by a two dimensional system:

$$ \dot{z}=f\left(z,y\right) $$

$$ \dot{y}=h(z)-y $$

which satisfies the following conditions at steady-state:

$$ f\left(z,h(z)\right)=0\ \mathrm{has}\ \mathrm{three}\ \mathrm{distinct}\ \mathrm{nonnegative}\ \mathrm{solutions}\ \mathrm{for}\ \mathrm{z}\ \mathrm{and} $$

$$ y=h(z)\ \mathrm{has}\ \mathrm{two}\ \mathrm{repeated}\ \mathrm{solutions}\ \mathrm{both}\ \mathrm{of}\ \mathrm{which}\ \mathrm{belong}\ \mathrm{to}\ \mathrm{the}\ \mathrm{stable}\ \mathrm{subspace}. $$

For example, the following ODEs meet the above conditions:

19 $$ \dot{Z}={f}_1\left(Z,Y\right)=-{Z}^3+6{Z}^2-11Z+6 $$

20 $$ \dot{Y}={f}_2\left(Z,Y\right)=-Y-{Z}^2+4Z+6 $$

**Fig. 6:**
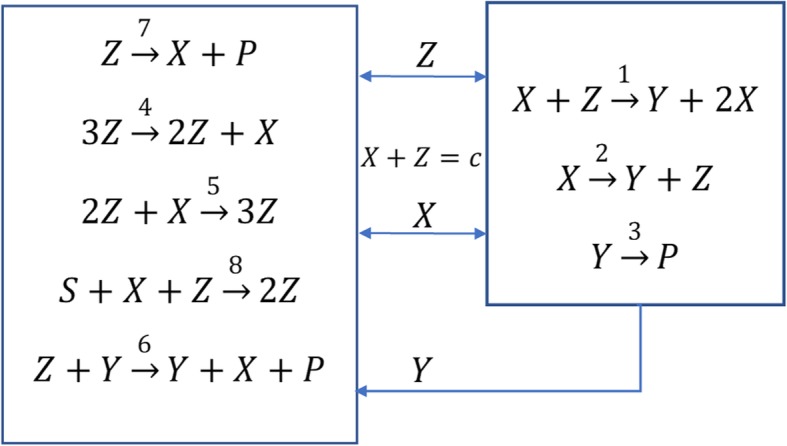
Example 3. *X* + *Z* = *c* (*constant*) is the conserved moiety

In this example we construct a reaction network that satisfies (19) and (20). Consider the reaction network shown in Fig. 6.

The conservation equations with mass action kinetics are given by two ODEs and one algebraic equation:

21 $$ \dot{Z}=-{k}_1 XZ+{k}_2X-{k}_4{Z}^3+{k}_5X{Z}^2-{k}_6 YZ-{k}_7Z+{k}_8 XZ $$

22 $$ \dot{Y}={k}_1 XZ+{k}_2X-{k}_3Y $$

23 $$ X+Z=c $$

Using the values for the rate constants given in Table 1 and eliminating *X* via (23*)* gives:

**Table 1:** Parameters for the reaction network of Example 3

*k*_1_	*k*_2_	*k*_3_	*k*_4_	*k*_5_	*k*_6_	*k*_7_	${k}_8^{\ast }$	c
1	1.1623	1	0.0156	1	0.0156	5.0983	0.1	5.1623

* constant concentration of the species S is lumped into ${k}_8=\overset{\sim }{k_8}S$

24 $$ \dot{Z}={f}_1\left(Z,Y\right)=-{Z}^3+6{Z}^2-11Z+6 $$

25 $$ \dot{Y}={f}_2\left(Z,Y\right)=-Y-{Z}^2+4Z+6 $$

The Gröebner basis *G* of (*f*_1_, *f*_2_) is calculated using the GroebnerBasis program under Polynomial Algebra of MATHEMATICA and the triangular system of basis polyniomials is given by (compare with Eq. 58):

26 $$ {g}_1(Y)=90-19Y+{Y}^2 $$

27 $$ {g}_2\left(Y,Z\right)=18-9Z-2Y+ ZY $$

28 $$ {g}_3\left(Y,Z\right)=-6-4Z+{Z}^2+Y $$

**Fig. 7:**
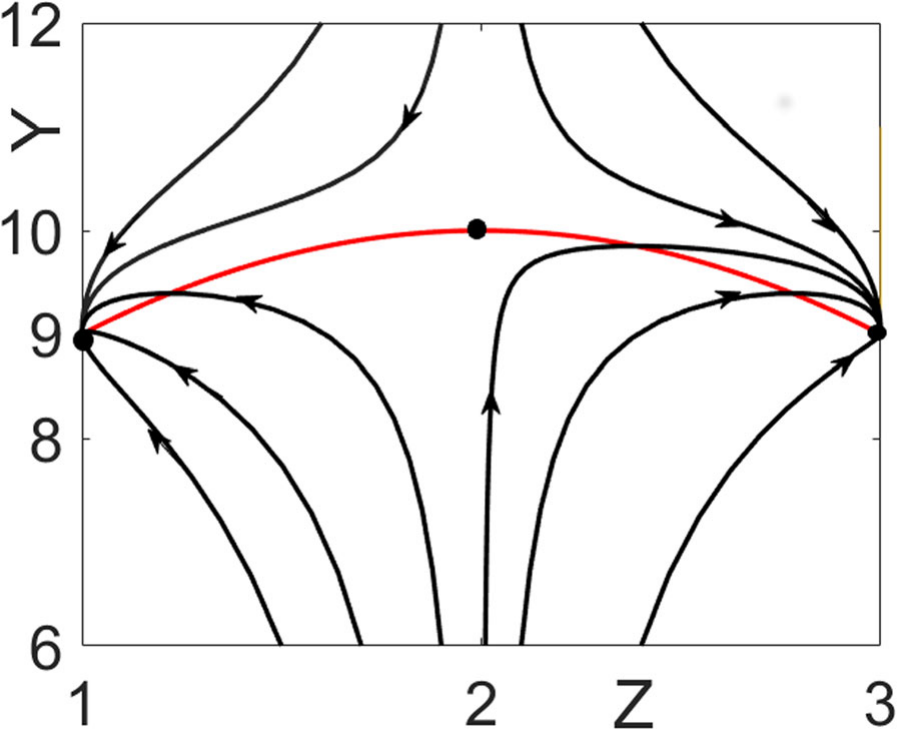
Bistable system with an output that does not switch at steady-state. Three steady-states are marked by black circles. The middle one at *Z* = 2, *Y* = 10 is the unstable steady-state. Trajectories starting from different initial conditions converge to the stable steady-state, where the output (*Y*) value does not change (i.e. *Y* = 9). The red curve is the production rate of *Y* which is the locus of steady-states for *Y* as a function of *Z*

Solving this triangular system of equations yields three distinct steady-state solutions for the state $$ x=\left(\begin{array}{c}Y\\ {}Z\end{array}\right) $$ = $$ \left(\begin{array}{c}9\\ {}1\end{array}\right),\left(\begin{array}{c}10\\ {}2\end{array}\right),\left(\begin{array}{c}9\\ {}3\end{array}\right) $$ as shown in Fig. 7. Therefore, the necessary condition *NC* for bistability stated in the Methods section (see Eq. 58) is satisfied. Next, one proceeds with the calculation of the Jacobians to establish sufficiency. Both eigenvalues of the Jacobian are negative at steady-states $$ \left(\begin{array}{c}9\\ {}1\end{array}\right),\left(\begin{array}{c}9\\ {}3\end{array}\right) $$ indicating that these are the stable steady-states; one eigenvalue is positive for $$ \left(\begin{array}{c}10\\ {}2\end{array}\right) $$ indicating that this is the unstable steady-state. Therefore, the system is bistable.

*g*_1_(*Y*) = 0 has two solutions, one less than the total number of steady-states for the state. Therefore, one of the roots *Y* = 9 is necessarily repeated in the steady-state solutions for the state: $$ x=\left(\begin{array}{c}Y\\ {}Z\end{array}\right) $$ = $$ \left(\begin{array}{c}9\\ {}1\end{array}\right),\left(\begin{array}{c}10\\ {}2\end{array}\right),\left(\begin{array}{c}9\\ {}3\end{array}\right) $$. Since the repeated roots belong to the stable steady-state solutions, the system is not switchable in the output *Y.* Figure 7 shows the state trajectories *x*(*t*) approaching the stable steady-states separated by the middle unstable fixed point at $$ \left(\begin{array}{c}10\\ {}2\end{array}\right) $$.

Figure 7 shows the state trajectories *x*(*t*) approaching the stable steady-states separated by the middle unstable fixed point at $$ \left(\begin{array}{c}10\\ {}2\end{array}\right) $$. However, unlike *Z* and *X = c-Z, Y* is not a switchable output since its value remains the same (equal to 9) at the stable steady-states.

The reason for *Y* not to be a switchable output can be physically explained as follows.

The mass balance for *Y* is determined by the following set of reactions (see Fig. 6):

$$ X+Z\overset{k_1}{\to }Y+2X $$

$$ X\overset{k_2}{\to }Y+Z $$

$$ Y\overset{k_3}{\to }P $$

and the constraint *X* + *Z* = *c.*

The first two reactions produce *Y* and the third reaction depletes *Y*. Conservation of *Y* is given by the following ODE:

29 $$ \dot{Y}={k}_1\left(c-Z\right)Z+{k}_2\left(c-Z\right)-{k}_3\mathrm{Y} $$

The first term *k*_1_(*c* − *Z*)*Z* is the rate of production of *Y* by the first reaction, and the second term *k*_2_(*c* − *Z*) is the rate of production of *Y* by the second reaction. The last term *k*_3_ *Y* is the rate of consumption of *Y* which is equal to the sum of the two production rates at steady-state:

30 $$ {k}_3\mathrm{Y}={k}_1\left(c-Z\right)Z+{k}_2\left(c-Z\right) $$

The total production rate of *Y* is maximum at the middle unstable steady-state as shown in Fig. 7. At the stable steady-state to the left of the maximum, the first production rate is greater than the second production rate *k*_1_(*c* − *Z*)*Z* > *k*_2_(*c* − *Z*) and the total production rate is 9. At the stable steady-state to the right of the maximum, the reverse is true i.e. *k*_1_(*c* − *Z*)*Z* < *k*_2_(*c* − *Z*) but the total production rate k_3_Y remains the same. Since *k*_3_ = 1 (see Table 1), *k*_3_Y = Y = 9 at the stable steady-states; thus, it cannot switch. The above result shows that, if an output species (*Y*) is produced by two reactions and the sum of the reactants is constant *X* + *Z* = *c*; then, for some values of the rate constants, the total production rate can remain the same at the stable steady-states leading to unswitchability of *Y* while both *X* and *Z* can switch.

**Fig. 8:**
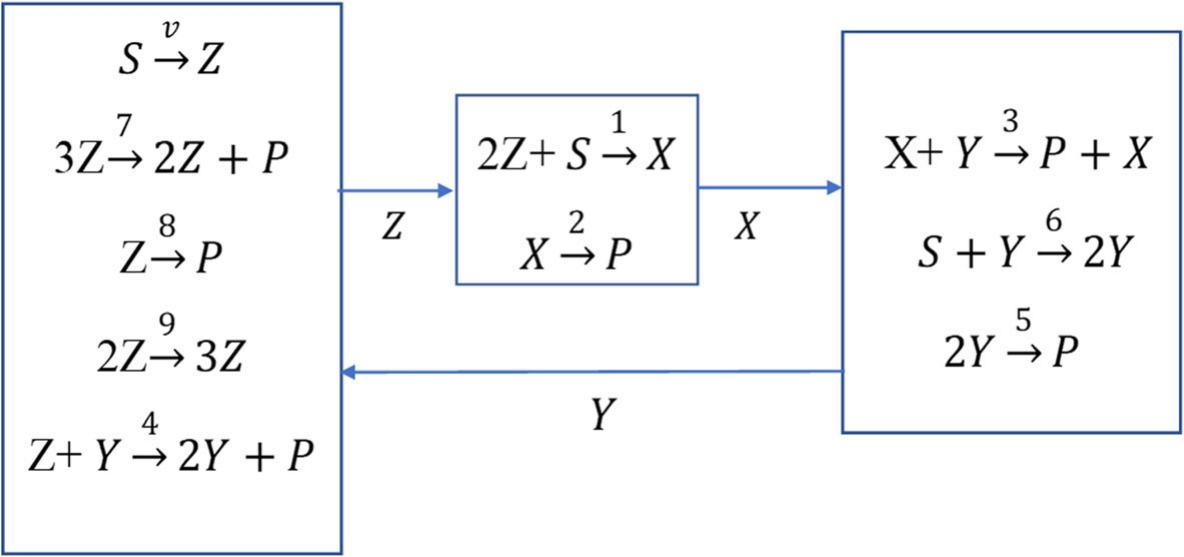
Reaction network for Example 4

**Example 4**. Consider the reaction network given in Fig. 8.

The conservation equations with mass action kinetics are given by:

31 $$ \dot{Z}=v-{k}_1{Z}^2-{k}_4 YZ-{k}_7{Z}^3-{k}_8Z+{k}_9{Z}^2={f}_1\left(Y,Z\right) $$

32 $$ \dot{X}={k}_1{Z}^2-{k}_2X={f}_2\left(X,Z\right) $$

33 $$ \dot{Y}=-{k}_3 XY+{k}_4 YZ-{k}_5{Y}^2+{k}_6Y=Y{f}_3\left(X,Y,Z\right) $$

The parameter values are given in Table 2.

**Table 2:** Parameters for the reaction network of Example 4

*k*_1_	*k*_2_	*k*_3_	*k*_4_	*k*_5_	*k*_6_	*k*_7_	*k*_8_	*k*_9_	*v*
1	1	0.166	1	0.166	1.667	2	16	16	24

At steady-state *Yf*_3_(*X*, *Y*, *Z*) = 0 and one solution is *Y* = 0, but for this value of *Y*, there is only one real positive solution for *Z* at 6.56; therefore, the system cannot be bistable. Thus, we consider the other solutions that satisfy *f*_3_(*X*, *Y*, *Z*) = 0. If *Y* is designated as the output variable, the univariate polynomial in *Y* does not exist. Choosing *Z* as the output variable and using the lexicographic order (*X*, *Y*, *Z*), the Gröbner basis is computed for the polynomials *f*_1_(*Y*, *Z*), *f*_2_(*X*, *Z*), *f*_3_(*X*, *Y*, *Z*) and the triangular system of basis polyniomials is given by

34 $$ {g}_1(Z)=24-26Z+9{Z}^2-{Z}^3=0 $$

35 $$ {g}_2\left(Z,X\right)=X-{Z}^2=0 $$

36 $$ {g}_3\left(Z,Y\right)=-Y-{Z}^2+6Z+10 $$

The steady-state solutions for the state are easily computed: $$ x=\left({x}_{ss}^{1,S},{x}_{ss}^{2,U},{x}_{ss}^{3,S}\right)=\left(\begin{array}{c}Z\\ {}X\\ {}Y\end{array}\right)=\left(\begin{array}{c}2\\ {}4\\ {}18\end{array}\right),\left(\begin{array}{c}3\\ {}9\\ {}19\end{array}\right),\left(\begin{array}{c}4\\ {}16\\ {}18\end{array}\right). $$ The system is bistable as determined by the Jacobians.

**Fig. 9:**
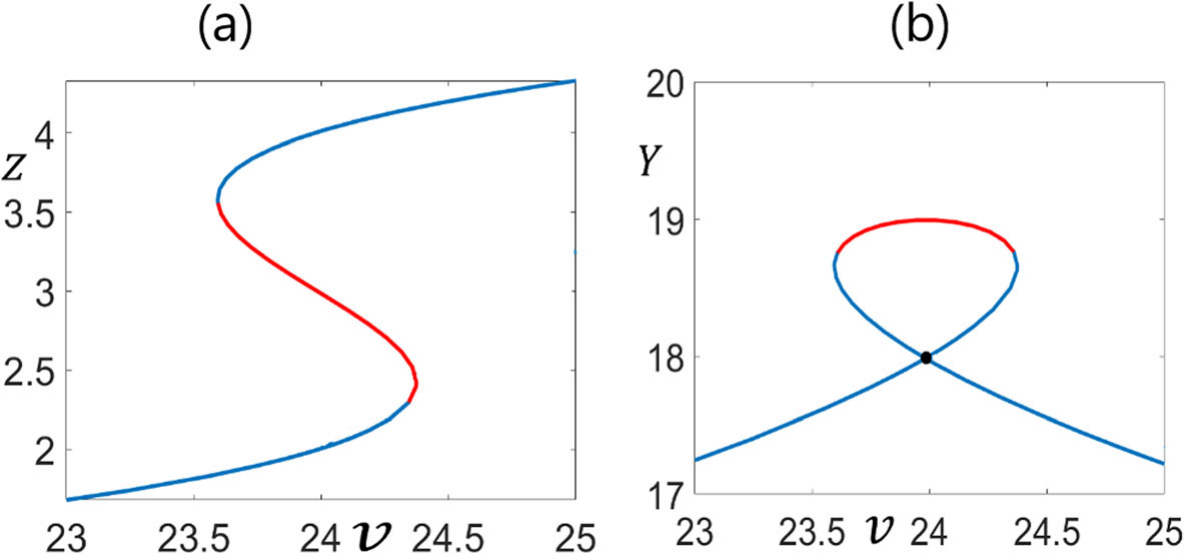
**a**. Bifurcation of *Z* with respect to *v*. Red and blue branches are the unstable and stable solutions. At *v* = 24, there are three distinct solutions for Z (stable, unstable, stable). **b**. Bifurcation of *Y* with respect to *v*. Red and blue branches are the unstable and stable solutions. At *v* = 24, there are two repeated stable solutions for Y at 18 (shown by black circle) and one unstable solution at 19

The univariate basis polynomial (34) satisfies the output switchability conditions; thus, the system is switchable in output *Z.* Due to (35) it is switchable in *X* as well. But it is not switchable in the output *Y,* since the solution *Y* = 18 determined from (36) is repeated in the stable steady-states. This is also shown in the bifurcation diagrams in Fig. 9.

c. *Cellular signaling pathways: AKT, RAS and MAPK signal transduction systems.*

**Example 5. AKT signaling pathway**

AKT signaling pathway plays a key role in the most significant metabolic action of insulin, which is the glucose uptake. Insulin resistance can develop through impairments in the signaling events involved in the activation of AKT. We use the following minimal dimensionless two-state model which we have derived earlier [39] from the original model presented in [6]:

37 $$ \dot{x_1}=-\frac{\beta {x}_1{x}_2}{K_1+{x}_1}+{k}_2\frac{E_2\left(1-{x}_1\right)}{K_1+\left(1-{x}_1\right)} $$

38 $$ \dot{x_2}=\left(\frac{\delta }{\beta}\right)\left(\frac{k_2}{k_1}\right){E}_2\ \lambda +\theta \left(1-{x}_1\right)-\updelta {x}_2 $$

where the states are the dimensionless concentrations *x*_1_ = pAKT (active AKT) and *x*_2_= pIRS (insulin receptor substrate). The input is the amount of insulin represented by *λ*. The parameter values are taken from [39].

$$ {k}_1={k}_2=0.909;\beta =1;\delta =1;{E}_2=1;{K}_1={K}_2=0.05;{d}_1=0.909,\theta =0.99 $$

Since the Gröebner basis is defined for polynomials, the right-hand side of (37) is first converted to a rational polynomial function so that (37) and (38) can be expressed as:

$$ \dot{\ {x}_1} $$= $$ \frac{f_1\left({x}_1,{x}_2\right)}{d_1\left({x}_1,{x}_2\right)} $$ and $$ \dot{\ {x}_2} $$ = *f*_2_(*x*_1_, *x*_2_)

where both *f*_1_(*x*_1_, *x*_2_) and *f*_2_(*x*_1_, *x*_2_) are polynomials. Since the steady-states are determined by the solutions *f*_1_(*x*_1_, *x*_2_) = 0 and *f*_2_(*x*_1_, *x*_2_) = 0, the Gröbner basis is computed for these two polynomials using MATHEMATICA, and for the insulin level *λ* =0.4 it is given by:

39 $$ {g}_1\left({x}_1\right)=0.0505-0.5146{x}_1+1.4439{x}_1^2-{x}_1^3 $$

40 $$ {g}_2\left({x}_1,{x}_2\right)=-1.39+0.99{x}_1+{x}_2 $$

Since *g*_1_(*x*_1_) given by (39) satisfies all the conditions for three distinct roots (Eqs. 59–61), the output *x*_1_ = *pAKT* is switchable, if the system is bistable. The necessary condition *NC* for bistability is satisfied since the triangular system (39)–(40) gives three steady-states:

$$ \left(\begin{array}{c}{x}_{1, ss}\\ {}{x}_{2, ss}\end{array}\right) $$ = $$ \left(\begin{array}{c}0.1689\\ {}1.2228\end{array}\right),\left(\begin{array}{c}0.310\\ {}1.083\end{array}\right),\left(\begin{array}{c}0.964\\ {}0.435\end{array}\right) $$

**Fig. 10:**
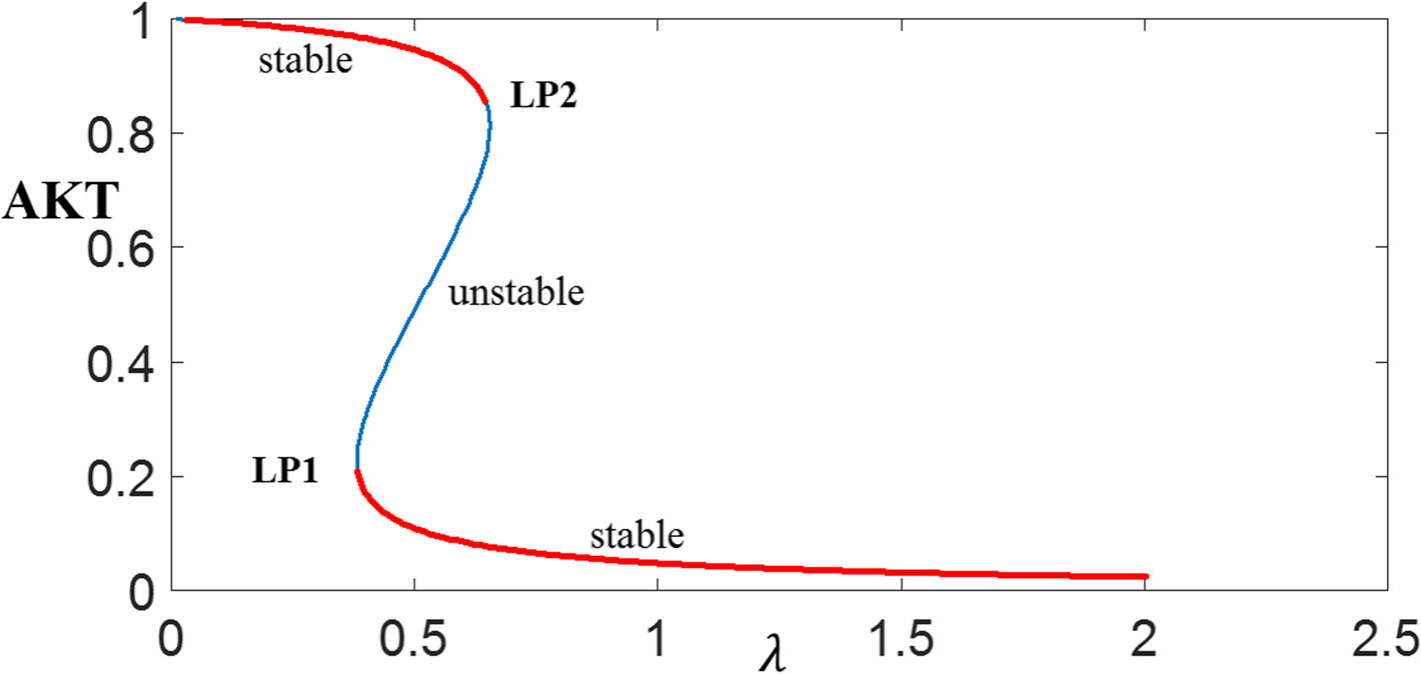
Bifurcation diagram for AKT showing the stable and unstable branches. LP1 and LP2 are the turning points. The bistable region is for *λ* between *LP*1 = 0.38 and *λ* = *LP*2 = 0.65

The sufficiency of bistability (i.e. checking the stability status of the three steady-states) is established by bifurcation analysis of eqs. (37)–(38) using XPPAUTO and is given in Fig. 10. The stable and unstable branches show that AKT is bistable for the range of *λ* between *LP*1 = 0.38 and *λ* = *LP*2 = 0.65. This confirms that the system is indeed bistable for *λ* = 0.4 and output *x*_1_ = *pAKT* is switchable.

In order for insulin to perform its function, AKT has to switch between its inactive and active states. Activated AKT (pAKT) enables the translocation of glucose transporter-4 (GLUT-4) from cytosol to the plasma membrane, thus glucose is taken into the cell.

**Fig. 11:**
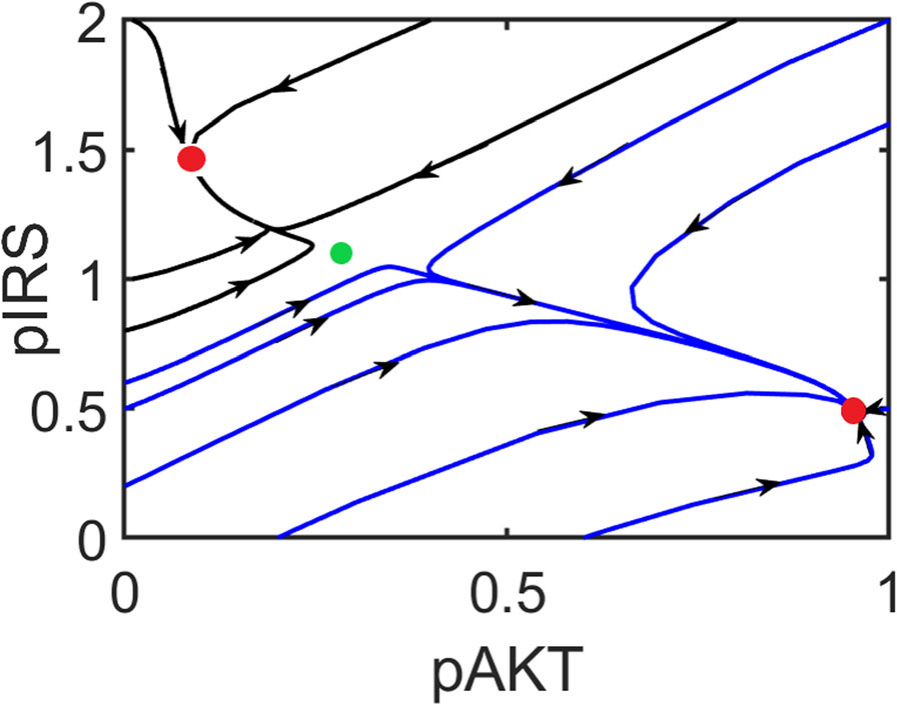
Bistability of the AKT signaling pathway. AKT system has two stable fixed points (red circles) and an unstable saddle-point (green circle). Trajectories are separated into two basins of attraction of the stable steady-states. *λ* = 0.4

The bistable behavior of pAKT is shown in Fig. 11. pAKT resides on either its active or inactive stable state depending on the initial condition.

**Fig. 12:**
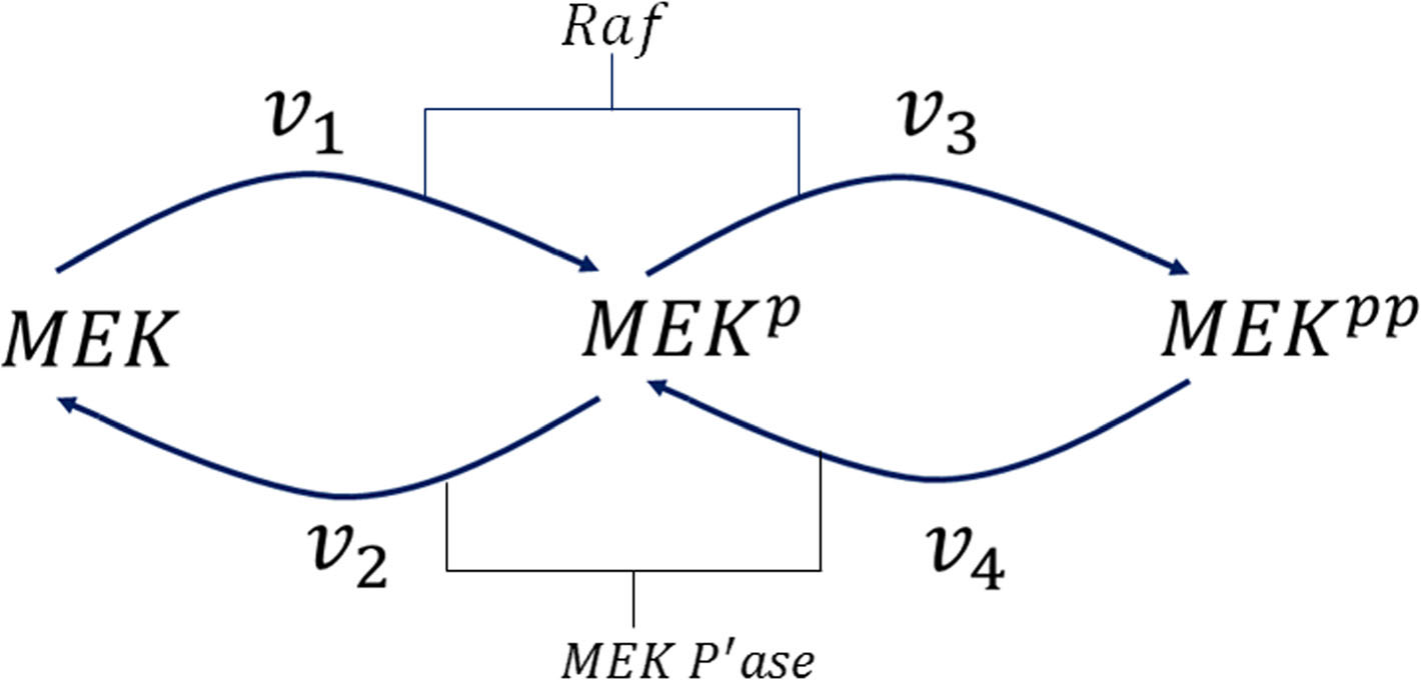
Dual phosphorylation/dephosphorylation cycle of protein MEK

The MAPK cascade is an integral part of the ERK (Extracellular Signal-Regulated Kinase) signaling pathway which plays a key role in cell cycle control. In the first stage of the cascade, RAF (Rapidly Accelerated Fibrosarcoma) gets activated by RAS-GTP (Rat Sarcoma Guanosine Triphosphate), and it triggers the second stage where MEK (Mitogen activated protein kinase kinase) gets double phosphorylated [2, 38]. This is followed by the activation ERK in the last stage. Here we will focus on the second stage which is shown in Fig. 12.

The two-site MAPK phosphorylation and dephosphorylation cycle with a distributive kinetic mechanism for the kinase and phosphatase possesses the necessary properties to exhibit bistable response [2, 40]. MEK and MEK^p^ compete for the same kinase (RAF) for phosphorylation; MEK^pp^ and MEK^p^ compete for the same phosphatase (MEK P’ase) for dephosphorylation. Through this competition, MEK inhibits the production of MEK^pp^, and MEK^pp^ inhibits the production of MEK. This double inhibition results in a positive feedback loop which leads to bistability under the right set of operating conditions or parameter values. Next we detect and confirm this bistability by using the method of Gröebner bases.

The model is taken from [40]:

41 $$ \dot{x} = {v}_2-{v}_1 $$

42 $$ \dot{y}={v}_3-{v}_4 $$

43 $$ 1-x-y-z=0 $$

$$ \left(x,y,z\right)\ \mathrm{are}\ \mathrm{the}\ \mathrm{dimensionless}\ \mathrm{concentrations}\ \frac{MEK}{M_T},\frac{MEK^{pp}}{M_T}\ \mathrm{and}\ \frac{MEK^p}{M_T} $$, (respectively)

*M*_*T*_ is the total concentration of *MEK*. The rates are given by:

$$ {v}_1=\frac{V_{m1}\frac{x}{K_{s1}}}{1+\frac{x}{K_{s1}}+\frac{z}{K_{s3}}}\ {v}_2=\frac{V_{m2}\frac{z}{K_{s2}}}{1+\frac{y}{K_{s4}}+\frac{z}{K_{s2}}} $$

$$ {v}_3=\frac{V_{m3}\frac{z}{K_{s3}}}{1+\frac{x}{K_{s1}}+\frac{z}{K_{s3}}}\ {v}_4=\frac{V_{m4}\frac{y}{K_{s4}}}{1+\frac{y}{K_{s4}}+\frac{z}{K_{s2}}} $$

with the parameters:

$$ {K}_{si}=\left({d}_i+{k}_i\right)/\left({M}_T\ast {a}_i\right)\ i=1:4 $$

$$ {V}_{mi}={k}_ic\ i=1,3 $$

$$ {V}_{mi}={k}_ip\ i=2,4 $$

where *c* and *p* are the concentrations of RAF kinase and the MEK phosphatase, respectively. Pertinent data is listed in Table 3.

**Table 3:** Parameter values for MAPK

*a*_1_	0.0204
*a*_2_	0.0493
*a*_3_	0.0564
*a*_4_	0.0326
*d*_1_	10.386
*d*_2_	2.716
*d*_3_	10.088
*d*_4_	0.813
*k*_1_	7
*k*_2_	11.13
*k*_3_	3.57
*k*_4_	1.13
*M*_*T*_	5128 *nM*
*c*	0.6 *nM*
*p*	1 *nM*

First (41)–(43) are re-expressed as:

44 $$ \dot{x}={v}_2-{v}_1=\frac{f_1\left(x,y,z\right)}{p_1\left(x,y,z\right)}\kern0.37em $$

45 $$ \dot{y}=\kern0.37em {v}_3-{v}_4=\frac{f_2\left(x,y,z\right)}{p_2\left(x,y,z\right)} $$

46 $$ 0=1-x-y-z={f}_3\left(x,y,z\right) $$

The Gröebner basis was obtained using MATHEMATICA:

$$ {g}_1(y)=-{y}^3+1.08{y}^2-0.1366y+4.54x{10}^{-4} $$

$$ {g}_2\left(x,y\right)=x-0.9142-15.99y+142.15{y}^2-132.66{y}^3 $$

$$ {g}_3\left(y,z\right)=z-0.085+16.99y-142.15{y}^2+132.66{y}^3 $$

which can be solved easily to give three solutions:

$$ \left(\begin{array}{c}y\\ {}x\\ {}z\end{array}\right)=\left(\begin{array}{c}0.0033\\ {}0.9655\\ {}0.0312\end{array}\right),\left(\begin{array}{c}0.1421\\ {}0.6969\\ {}0.1610\end{array}\right),\left(\begin{array}{c}0.9347\\ {}0.0065\\ {}0.0588\end{array}\right) $$. Evaluation of the eigenvalues of the Jacobians shows that the system is bistable. Since the univariate basis polynomial *g*_1_(*y*) meets the conditions for three distinct roots, the system is switchable for the output

$y=\frac{MEK^{pp}}{M_T}$as well. In fact, all the states are switchable.

Example 7. RAS signaling

RAS, which is a small GTP (Guanosine Triphosphate) binding protein, serves as an important molecular switch in signaling pathways. For example, in ERK signaling pathway, RAS interacts with the ShC-Grb2-SOS complex, and it is transformed to its active conformation by exchanging GDP (Guanosine Diphosphate) for GTP. Active Ras-GTP starts the sequential phosphorylation of the MAPK pathway that consists of the RAf-MEK-ERK signaling cascade. Catalytic activation of RAS by the SOS (Son of Sevenless) complex Shc-Grb2-SOS while RAS-GTP is bound to its allosteric site creates a positive loop resulting in a bistable switching response of Ras-GTP [41]. The model is taken from [41] and it is converted to a dimensionless form. It consists of the following equations:

47 $$ \dot{\left[{R}_T\right]}=-{\beta k}_2\left[S\right]\left[{R}_T\right]+{k}_{-2}\left[S{R}_T\right]+\frac{k_4^{cat}\alpha \left[{R}_D\right]\left[S{R}_D\right]}{K_{4m}+\beta \left[{R}_D\right]}+\frac{k_3^{cat}\alpha \left[{R}_D\right]\left[S{R}_T\right]}{K_{3m}+\beta \left[{R}_D\right]}-\frac{k_5^{cat}\left[{R}_{GAP}\right]\left[{R}_T\right]}{K_{5m}+\beta \left[{R}_T\right]} $$

48 $$ {\displaystyle \begin{array}{c}={f}_1\left(\left[S\right],\left[{R}_T\right],\left[{R}_D\right],\left[S{R}_T\right],\left[S{R}_D\right]\right)/{d}_1\left(\left[S\right],\left[{R}_T\right],\left[{R}_D\right],\left[S{R}_T\right],\left[S{R}_D\right]\right)\\ {}\dot{\left[S{R}_T\right]}=\beta {k}_2\left[S\right]\left[{R}_T\right]-{k}_{-2}\left[S{R}_T\right]={f}_2\left(\left[S\right],\left[\ {R}_T\right],\left[S{R}_T\right]\right)\end{array}} $$

49 $$ \dot{\left[S{R}_D\right]}=\beta {k}_1\left[S\right]\left[{R}_D\right]-{k}_{-1}\left[S{R}_D\right]={f}_3\left(\left[S\right],\left[{R}_D\right],\left[S{R}_D\right]\right) $$

50 $$ 1-\left[{R}_D\right]-\left[{R}_T\right]-\left(\frac{\alpha }{\beta}\right)\left[{SR}_D\right]-\left(\frac{\alpha }{\beta}\right)\left[{SR}_T\right]={f}_4\left(\left[{R}_T\right],\left[{R}_D\right],\left[{SR}_D\right],\left[{SR}_T\right]\right)=0 $$

51 $$ 1-\left[S\right]-\left[{SR}_D\right]-\left[{SR}_T\right]={f}_5\left(\left[S\right],\left[{SR}_D\right],\left[{SR}_T\right]\right)=0 $$

The variables are defined as follows: ***S*** is the Shc-Grb2-SOS complex; ***R***_***T***_ is RAS-GTP; ***R***_***D***_ is RAS-GDP, ***SR***_***D***_ and ***SR***_***T***_ are the complexes formed by the reactions; [.] denotes the concentration. The total concentration of *S* molecules is ***α***, and the total concentration of RAS molecules is ***β.*** The values for the parameters are given in Table 4.

**Table 4:** Parameter values for RAS

*β*	200 *nM*
*α*	10 *nM*
*k*_1_	1.125*e* − 4 *nM*^−1^ *s*^−1^
*k*_2_	1.0625*e* − 4*nM*^−1^ *s*^−1^
*k*_−1_	3*s*^−1^
*k*_−2_	0.4*s*^−1^
${k}_3^{cat}$	1.75*s*^−1^
${k}_4^{cat}$	0.003*s*^−1^
${k}_5^{cat}$	0.1*s*^−1^
*RGAP*	0.1 *nM*
*K*_3*m*_	2.7388e3 *nM*
*K*_4*m*_	1.52304e4*nM*
*K*_5*m*_	17.869*nM*

**Fig. 13:**
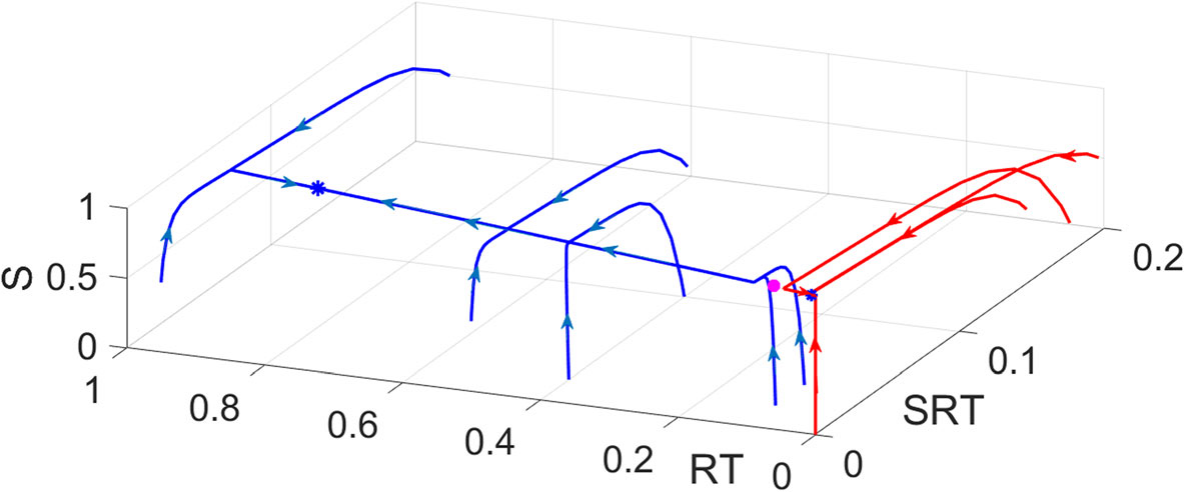
Bistability of a three-dimensional RAS model. The system has two stable fixed points (blue stars) and an unstable saddle-point (magenta circle). Trajectories reach the stable fixed points after following the unstable manifold of the saddle point. *S* is the Shc-Grb2-SOS complex. *R*_*T*_ is RASGTP. *SR*_*T*_ is the SOS-RASGTP complex

The model has three steady-states, two of which are stable representing the active and inactive states of RAS, and a saddle point with a positive eigenvalue. Bistability is illustrated in Fig. 13. Trajectories first converge to the unstable manifold, and then they are attracted to either of the two steady-states.

MATLAB computes the univariate basis polynomial as a cubic polynomial:

$$ {g}_1\left({R}_T\right)=-{R_T}^3+0.912{R_T}^2-0.07{R}_T+4.05x{10}^{-6} $$

with three distinct roots, thus *R*_*T*_ is a switchable output.

## Discussion

We have developed a new method to detect and analyze biological switches by simultaneously treating bistability and output switchability using the Gröebner bases. As demonstrated by several examples, the proposed methodology offers the following:

- First the method provides computational advantages due to its nice properties. Specifically, the method of Gröebner bases to solve polynomial systems can be seen as a multivariate, non-linear generalization of the Gaussian elimination for linear systems. Multistationarity is easily checked by solving a triangular set of equations which facilitates the root finding. Bistability is confirmed by local stability analysis using the Jacobian which is also straightforward.
- It provides a theoretical framework and a systematic methodology that analyzes both bistability and output switchability simultaneously. Output switchability conditions follow immediately from the univariate Gröebner polynomial basis and are easy to check. We show by Examples 3 and 4 that some bistable systems can have outputs that do not switch their steady-states.
- The univariate Gröebner basis polynomial provides useful biological insight which can help in the design of biological switches. A bistable dynamical system with output x, is output switchable, if its univariate basis polynomial g_1_(x) =  − x^3^ + bx^2^ − cx + d with b > 0, c > 0, d ≥ 0 has three distinct nonnegative roots. This result provides some biological insight. The terms −x^3^ − cx represent the rate of depletion of species x, and the terms bx^2^ + d represent the rate of production of species x. This suggests that a biological switch for an output species x can be designed by constructing a reaction network (with its corresponding ODEs) whose univariate Gröbner basis polynomial in x has the above types of depletion and production terms. In fact Examples 1, 3 and 4 were constructed in this fashion.

## Conclusions

We have presented a new method to detect biological switches by analyzing their bistability and output switchability properties. The methodology is based on the Gröebner bases. Conditions are established to make the connections between the Gröebner bases, bistability and output switchability. Various examples are given to elucidate the theoretical results. We show that the method can analyze bistability and output switchability while providing useful insight into the underlying mechanisms. The method is easy to apply since significant software such as MAPLE, MATLAB and MATHEMATICA exists to perform the Gröebner bases computation.

It goes without saying that high dimensionality can pose computational problems as in other methods. As a remedy, techniques such as lumping, network complexity reduction can be used to reduce the number of ODEs before the Gröebner basis calculation is carried out. In general the method can be applied to other types of polynomial differential equations derived from data instead of first principles. Such potential models include the nonlinear polynomial regression models.

## Methods

Bistability and output switchability analysis is based on the Gröebner bases.

### The Gröebner bases

Any finite set of multivariate polynomials *F* can be transformed by an algorithm (see Buchberger’s algorithm [26]) into another set of basis polynomials, called the Gröebner basis *G*. Many problems that are difficult to handle by the original set of polynomials can be easily solved by using the method of Gröebner bases due to its “nice” properties. Readily available computer software such as MAPLE, MATHEMATICA and MATLAB are equipped with the computational machinery of the Gröebner bases. The most basic definitions and properties of the Gröebner bases are presented in the Additional file 1. In this paper we explore the Gröebner bases within the context of bistability analysis. To this end, we state some of the useful properties of the Gröebner bases for solving polynomial equations.

### Solution of polynomial equations by the Gröebner bases

The method of Gröebner bases to solve polynomial systems can be seen as a multivariate, non-linear generalization of the Gaussian elimination for linear systems [42].

The *ideal I* =  < *f*_1_, *f*_2_, . . , *f*_*n*_> is the set of all possible linear combinations of *f*_*i*_ ′ *s* where the coefficients are polynomials *p*_*i*_ (Additional file 1). Since *F* = (*f*_1_, *f*_2_, , .., *f*_*n*_) and its Gröebner basis *G* generate the same ideal, they have the same solutions [42, 43]. The advantageous property of the Gröebner basis *G* is that it yields a triangular system which conveniently seperates the variables and drastically simplifies the calculation. This triangular system is like the reduced row echelon form obtained by pivoting in Gaussian elimination in the case of linear systems.

Consider the steady-state solutions of the dynamical system (Eqs. 4–5) which satisfy following set of polynomial equations:

52 $$ {f}_1=16y-{x}^2- xy-1.5x=0 $$

53 $$ {f}_2={x}^2-8y=0 $$

The Gröebner basis *G* for these two polynomials with respect to the *lexicographic ordering* is given in a triangular form:

54 $$ {g}_1(x)=12x-8{x}^2+{x}^3 $$

55 $$ {g}_2\left(x,y\right)=-0.125{x}^2+y $$

First the univariate basis polynomial *g*_1_(*x*) is easily solved for its roots *x*: (0, 6,2). Next these *x* values are substituted into the bivariate basis *g*_2_(*x*, *y*) to determine its corresponding roots *y*: (0,4.5,0.5). Thus, the solutions (*x*, *y*) of the original set of polynomials *F* are obtained: (0, 0), (6,4.5) and (2,0.5).

### Detection of Bistability

Consider the dynamical system *S*_*f*_ given by:

56 $$ \dot{x}=f(x)\ \mathrm{with}\ x={\left[{x}_1{x}_2\dots ..{x}_n\right]}^T $$

where the steady-state solutions satisfy *f*(*x*) = 0, and they are denoted as

${x}_{ss}^i={\left[{x}_{1, ss}^i\ {x}_{2, ss}^i\dots \dots .{x}_{n, ss}^i\ \right]}^T\ i=1:m$ with *m* the number of solutions.

Let *G* = [*g*_1_(*x*_1_), *g*(*x*)] be the Gröebner basis for the ideal *I* =  < *f*_1_, *f*_2_, …, *f*_*n*_>, where *g*_1_(*x*_1_) is the univariate basis polynomial, and *g*(*x*) is the vector of remaining polynomials arranged in triangular form:

57 $$ g(x)=\left[{g}_2\left({x}_1,{x}_2\right)\ {g}_3\left({x}_1,{x}_2,{x}_3\right)\dots ..{g}_t\left({x}_1,{x}_2,{x}_3,..,{x}_n\right)\right]. $$

#### A necessary condition for bistability (NC)

The dynamical system *S*_*f*_ is bistable only if the following set of equations have three distinct real nonnegative solutions $\left({x}_{ss}^1,{x}_{ss}^2,{x}_{ss}^3\right)$ for the state *x*:

58 $$ {\displaystyle \begin{array}{c}{g}_1\left({x}_1\right)=0\\ {}{g}_2\left({x}_1,{x}_2\right)=0\\ {}\begin{array}{c}{g}_3\;\left({x}_1,{x}_2,{x}_3\right)=0\\ {}\begin{array}{c}.\\ {}\ {g}_t\left({x}_1,{x}_2,{x}_3,..,{x}_n\right)=0\end{array}\end{array}\end{array}} $$

Necessity follows from the working definition of bistability which requires three distinct steady-state solutions for the state vector *x*, and the fact that the steady-state solutions of *S*_*f*_ and the solutions of the Gröebner basis polynomials (58) are the same. Moreover, for a zero-dimensional ideal, the triangular structure in the Gröebner basis always exists [42]. For Gröbner bases, unlike other triangular systems, it is guaranteed that each partial solution can be extended to a full solution. This means that every solution *x*_1_ of the first polynomial can be extended to a solution (*x*_1_, *x*_2_) of the polynomials in *x*_1_ and *x*_2_, and each of these solutions can be further extended to a solution (*x*_1_, *x*_2_, *x*_3_) of the polynomials in *x*_1_, *x*_2_, *x*_3_, etc.

It is important to note that bistability cannot be detected by checking the number of solutions of the univariate basis polynomial *g*_1_(*x*_1_) alone but the whole basis must be considered. This follows from the fact that the number of solutions of *g*_1_(*x*_1_) = 0 can be less than the number of solutions for the state *x.*

Systems that fail to meet the necessary condition cannot be bistable; thus, they are easily eliminated from further consideration. However, satisfying the necessary condition does not guarantee bistability. Further tests should be applied to confirm it. The most common approach is to compute the eigenvalues of the Jacobian of *f*(*x*). The Jacobian matrix is obtained by linearizing the dynamical system *S*_*f*_ at its steady-states ${x}_{ss}^i$:

$$ {J}^i=\frac{\partial f(x)}{\partial {x}^T}\left|\begin{array}{c}\\ {}{x}_{ss}^i\end{array}\right. $$

A steady-state ${x}_{ss}^i$ of the dynamical system *S*_*f*_ is stable if all the eigenvalues of Jacobian matrix *J*^*i*^ have negative real parts. The steady-state is unstable if at least one of the eigenvalues has a positive real part [44]. Bistability can be easily ascertained by checking the stability status of each of the three distinct steady-states $\left({x}_{ss}^1,{x}_{ss}^2,{x}_{ss}^3\right)$ of *S*_*f*_.

### Detection of switchable outputs

A bistable dynamical system with output $$ {x}_1,{S}_{f,{x}_1}, $$ is output switchable, if its univariate basis polynomial satisfies the following conditions:

59 $$ {g}_1\left({x}_1\right)=-{x_1}^3+b{x_1}^2-c{x}_1+d $$

60 $$ b>0,c>0,d\ge 0. $$

61 $$ D=-27{d}^2+18 bcd-4{c}^3-4{b}^3d+{b}^2{c}^2>0 $$

**Fig. 14:**
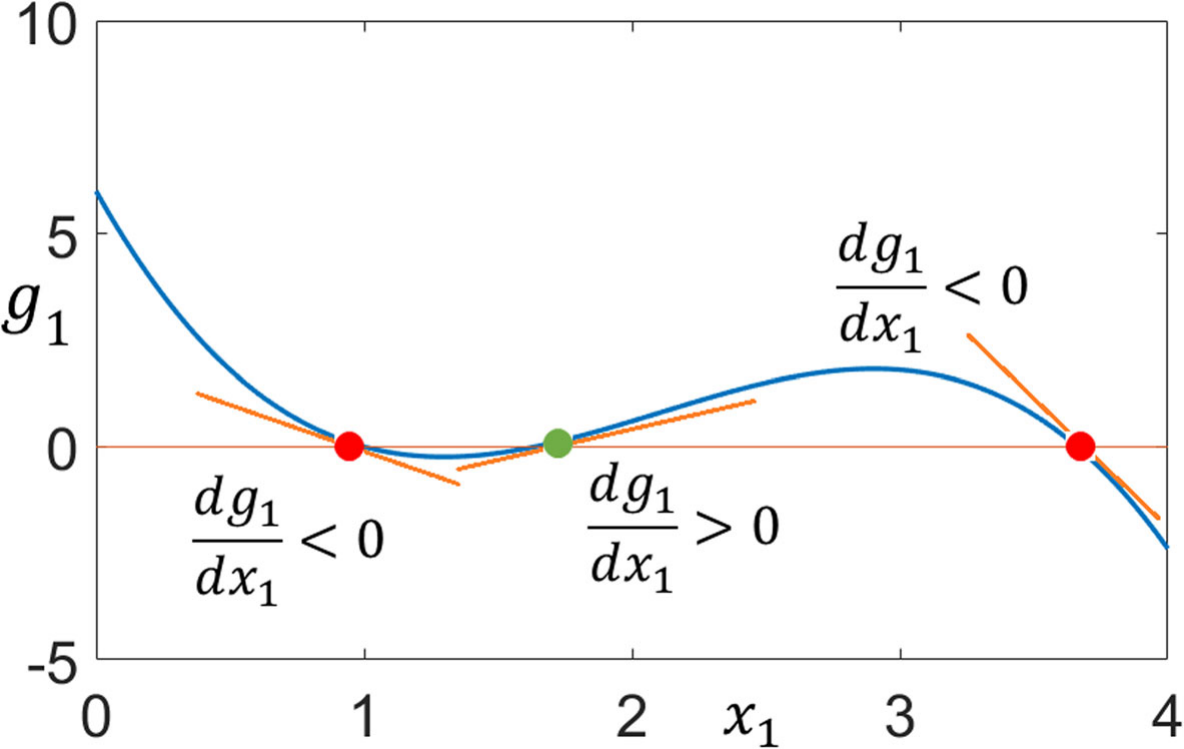
The univariate basis poynomial. Distinct roots are shown by the circles (red for stable; green for unstable). Slopes of the tangent lines (Jacobians) are negative and positive at the stable fixed points and saddle-point, respectively

These conditions guarantee that *g*_1_(*x*_1_) has three distinct nonnegative roots so that, the output *x*_1_ can take different values when the state *x* changes between its stable steady-states. Existence of three distinct nonnegative roots can be shown as follows. According to the Descartes’ rule of signs, the maximum number of negative real roots of a polynomial *f*(*x*) is equal to the number of changes in sign of the coefficients of the terms of *f*(−*x*). When (60) is true, there are no sign changes in the coefficients of *g*_1_(−*x*_1_) in (59); thus, *g*_1_(*x*_1_) can have maximum three nonnegative roots. The inequality (61) is the cubic discriminant condition which guarantees that there are three distinct real roots. The general graph of *g*_1_(*x*_1_) satisfying conditions (59)–(61) is shown in Fig. 14.

If the univariate basis polynomial *g*_1_(*x*_1_) does not have three distinct roots; then, the bistable system ${S}_{f,{x}_i}$ is switchable in its output *x*_*i*_, only if one of the repeated solutions belongs to the unstable steady-state solution ${x}_{ss}^{2,U}$ . This follows from the definition of output switchability which requires that the output values are different at the two stable steady-states.

It is possible that for an output variable of interest y, the univariate basis polynomial *g*_1_(*y*) may not exist. If this happens, by changing the lexicographic ordering, the triangular system of basis polyniomials (58) is calculated using a different univariate basis polynomial *g*_1_(*x*_*i*_) where *x*_*i*_ ≠ *y*. In such cases, the bistable system is switchable in its output *y*, only if the solutions *y* obtained from the triangular system of basis polynomials are different at the two stable steady-states (${x}_{ss}^{1,S}\  and\ {x}_{ss}^{3,S}\Big)$.

### The univariate basis polynomial dynamics

For a bistable system with a switchable output *x*_1_, we define the univariate basis polynomial dynamics describing the output as:

62 $$ {S}_{g_1,{x}_1}:\dot{x_1}={g}_1\left({x}_1\right)=-{x_1}^3+b{x_1}^2-c{x}_1+d $$

${S}_{g_1,{x}_1}$ is bistable as seen from the signs of the local Jacobians depicted in Fig. 14. Note that the output trajectories of ${S}_{g_1,{x}_1}\ \mathrm{and}$ the original system ${S}_{f,{x}_1}$ are different in transient but both converge to the same stable steady-state, since they have the same output solutions due to the property of Gröbner basis. When the univariate basis polynomial has three distinct roots, it is always possible to construct the one-dimensional bistable system ${S}_{g_1,{x}_1}.$

It can be seen from Eq. (62) that |*g*_1_(*x*_1_)| is the imbalance between the rate at which the output species is generated and the rate at which it is depleted:

63 $$ {x}_1={g}_1\;\left({x}_1\right)=\left(b{x_1}^2+d\right)-\left({x}_1^3+c{x}_1\right)={g}_{generation}\;\left({x}_1\right)-{g}_{generation}\;\left({x}_1\right) $$

**Fig. 15:**
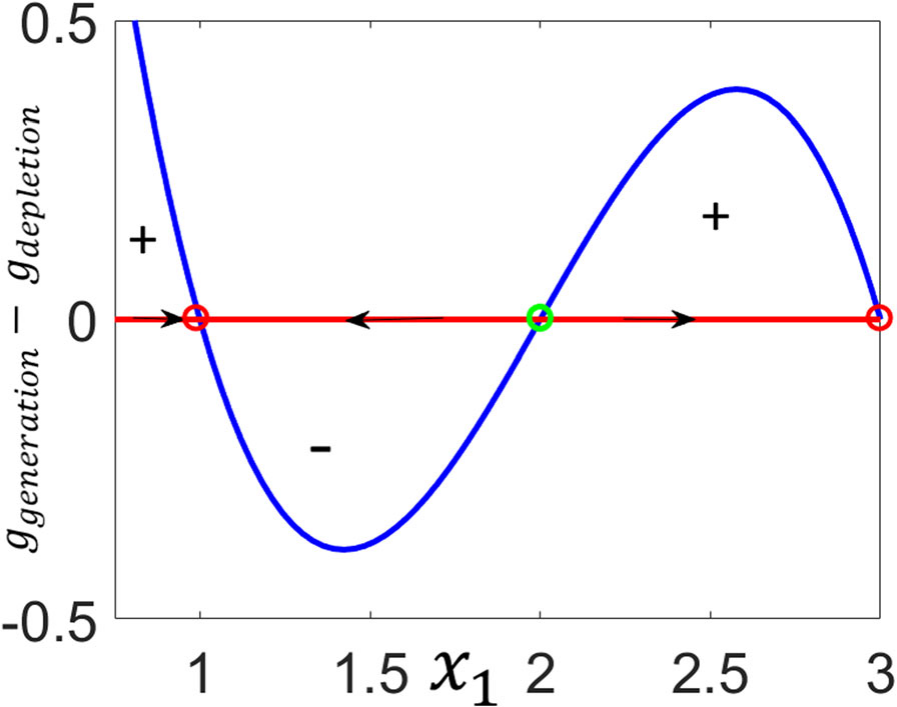
Bistable univariate dynamical system. Depending on the initial sign of *g*_*generation*_ − *g*_*depletion*_, trajectories approach either of the stable steady-states where. *g*_*generation*_ = *g*_*depletion*_.

At the roots of *g*_1_(*x*_1_), the two rates equilibrate. Between the roots, the sign of (*g*_*generation*_ − *g*_*depletion*_) alternates as (+ − +) to create a bistable switching output response as shown in Fig. 15.

According to (63) for a bistable system to have a switchable output *x*_1_, the rate of generation must consist of a quadratic and a constant term, and the rate of depletion must consist of a cubic and a linear term:

64 $$ {g}_{generation}=\left|b\right|{x_1}^2+\left|d\right| $$

65 $$ {g}_{depletion}={x_1}^3+c{x}_1 $$

This suggests that a biological switch for an output species can be synthetically designed by constructing a reaction network (and its corresponding ODEs) who’s univariate Gröbner basis polynomial has the above types of depletion and production terms. Examples 1, 3 and 4 were constructed in this fashion.

### Analysis procedure

We have incorporated the theoretical results into the following analysis procedure:

1. Given a Differential Algebraic System, compute its Gröbner basis. We have used MATLAB and MATHEMATICA for this purpose.
2. By solving the triangular system of eqs. (58), check if three distinct nonnegative solutions exist in some region of the state space. If it does, check the eigenvalues of the Jacobian at the three steady-states to decide if the system is bistable. If the system is bistable, proceed to the next step. If three distinct nonnegative solutions for the state do not exist, the system cannot be (fixed-point) bistable and stop.
3. For the bistable system, identify the output of interest *y* and compute the univariate basis polynomial in *y* i.e. *g*_1_(*y*) by reordering the variables, if necessary. Compute the roots of *g*_1_(*y*). If the number of roots is three, the bistable system is switchable in the output if these roots are all distinct. In all other cases where there are two repeated roots, one of the repeated roots must belong to the unstable steady-state solution ${x}_{ss}^{2,U}$. Otherwise the system is not switchable in output *y.*
4. In case the univariate basis polynomial *g*_1_(*y*) does not exist, solutions *y* are calculated using a different univariate basis polynomial *g*_1_(*x*_*i*_) where *x*_*i*_ ≠ *y*. The bistable system is switchable in its output *y*, only if the solutions at two stable steady-states (${x}_{ss}^{1,S}\  and\ {x}_{ss}^{3,S}\Big)$ are distinct.

## Supplementary information

**Additional file 1.** Background on Gröebner Bases [42, 43].

## Data Availability

All data generated or analysed during this study are included in this manuscript. Given the data, the Gröbner bases were calculated by calling the *GroebnerBasis* routine available in MATHEMATICA or the *gbasis* routine available in the Symbolic Math Toolbox of MATLAB. No special software is needed except to enter the data reported in the manuscript in MATLAB or MATHEMATICA.

## Abbreviations

AKT: Protein kinase B
CRN: Chemical Reaction Network
DAE: Differential Algebraic Equations
ERK: Extracellular Signal-Regulated Kinase
GDP: Guanosine Diphosphate
GLUT-4: Glucose transporter-4
Grb2: Growth factor receptor-bound protein 2
GTP: Guanosine Triphosphate
ICSA: Instability Causing Structure Analysis
IRS: Insulin receptor substrate
LP: Limit Point
MAPK: Mitogen activated protein kinase
MEK: Mitogen activated protein kinase kinase
NC: Necessary condition
ODE: Ordinary Differential Equations
pAKT: Phosphorylated AKT
pIRS: Phosphorylated Insulin Receptor Substrate
RAF: Rapidly Accelerated Fibrosarcoma
RAS: Rat Sarcoma
RAS-GTP: Active form of RAS
RGAP: RAS GTPase Activating Proteins
Shc: Src homology 2 domain containing
SOS: Son of Sevenless
