# Supplementary material for: Detection of biological switches using the method of Gröebner bases
Source: BMC Bioinformatics. 2019 Nov 28;20:615. doi: 10.1186/s12859-019-3155-0 (PMC6883700; doi:10.1186/s12859-019-3155-0)
Supplement: Supplementary file 1 — Additional file 1. Background on Gröebner Bases [42, 43]. [file 12859_2019_3155_MOESM1_ESM.docx]

**Additional file 1. Background on Gröebner Bases [42, 43].**

Let $k$ be a field e.g fields of real, complex and rational numbers. A [polynomial](http://www.wiki-zero.co/index.php?q=aHR0cHM6Ly9lbi53aWtpcGVkaWEub3JnL3dpa2kvUG9seW5vbWlhbA) is a sum $c_{1}M_{1}+c_{2}M_{2}+\ldots+c_{n}M_{n}$ {\displaystyle c_{1}M_{1}+\cdots +c_{m}M_{m}}where the {\displaystyle c_{i}}${}c_{i}\in$*k* and {\displaystyle M_{i}}${}M_{i}'s$ are [monomials](http://www.wiki-zero.co/index.php?q=aHR0cHM6Ly9lbi53aWtpcGVkaWEub3JnL3dpa2kvTW9ub21pYWw) or power products. Each monomial $M_{i}$ is a power product $x_{1}^{a_{1}}\ldots x_{n}^{a_{n}}${\displaystyle M=x_{1}^{a_{1}}\cdots x_{n}^{a_{n}},} with{\displaystyle a_{i}}$a{}$ nonnegative integers $a_{i}$. $k[x_{1},x_{2},\ldots,x_{n}]$

denotes the set of all polynomials in *n* variables with coefficients in the field *k*.

The leading power product (lp) of a polynomial is the largest power product appearing in the polynomial. For this, some ordering has to be imposed on the power products. The most common ordering is the *lexicographic order,* which for three variables looks like:

$$1<x<x^{2}<..<y<xy<x^{2}y<x^{3}y<..<y^{2}<{xy}^{2}<{x^{2}y}^{2}<x^{3}y^{2}<\ldots.$$

*Reduction of a polynomial*: This is the division process for a polynomial with respect to a basis. The power products in a polynomial are replaced by power products that are smaller in the chosen ordering by subtracting the multiples of basis elements from the polynomial. This is repeated until division is no longer possible.

Example . Divide$f=x^{2}y+xy^{2}+y^{2}$ by the basis polynomials $B: (f_{1}=xy-1$, $f_{2}=y^{2}-1$). Eliminating variables in the *lexicographic order*$1<x^{2}<y<xy<x^{2}y<y^{2}<{xy}^{2}$ , the three reduction steps are:

$$x^{2}y+xy^{2}+y^{2}\overset{\to}{xy-1}x^{2}y+y^{2}+y\overset{\to}{y^{2}-1}x^{2}y+y+1\overset{\to}{xy-1}y+x+1$$

In the first step the polynomial on the right is obtained from the polynomial on the left by subtraction of $the first basis element xy-1$ times y. This replaces the monomial $xy^{2}$ by the smaller term *y*. Division stops when the last polynomial, $y+x+1$, cannot be reduced further. Thus, it is called the remainder of *f*  upon division by *B* i.e*.* $R\left( f,B \right).$

The above division gives:

$$x^{2}y+xy^{2}+y^{2}=y\left( xy-1 \right)+1\left( y^{2}-1 \right)+x\left( xy-1 \right)+\left( y+x+1 \right)$$

$f=\left( x+y \right)f_{1}+(1{)f}_{2}+R\left( f,B \right)$

The *ideal* generated by the polynomials $(f_{1},f_{2},\ldots.., f_{s})$ is the set of all possible linear combinations of $f_{i}'s$ where the coefficients are polynomials $p_{i}$ . It is denoted by

$I=<f_{1},f_{2},..,f_{s}>$=$\{ \sum_{i=1}^{s} p_{i}f_{i} s.t. p_{i}\epsilon k\left[ x_{1},x_{2},\ldots,x_{n} \right], i=1,2..,s\}$.

Let *K* be an extension field *k,* that is, *K* is a field such that $k\subseteq K.$ Given a subset

S$\subseteq k\left[ x_{1},x_{2},\ldots,x_{n} \right],$ a variety $V_{K}\left( S \right)\mathrm{in} K^{n}$ is defined by

$V_{K}\left( S \right)=\left\{ (a_{1},\ldots,a_{n} \right.)\epsilon K^{n}$ | $f\left( a_{1},\ldots,a_{n} \right)=0 for all f\epsilon S\}$

An ideal I is zero­ dimensional if the associated variety V (I) is a finite set.

A Gröebner basis belongs to a particular kind of [generating set of an ideal](http://www.wiki-zero.co/index.php?q=aHR0cHM6Ly9lbi53aWtpcGVkaWEub3JnL3dpa2kvSWRlYWxfKHJpbmdfdGhlb3J5KSNJZGVhbF9nZW5lcmF0ZWRfYnlfYV9zZXQ) with “nice” properties and it is defined as follows.

*Definition*: A set of non-zero polynomials $G=(g_{1},g_{2},..,g_{t})$ contained in an ideal *I*, is called a Gröebner basis if and only if for all $f\epsilon I$ such that $f\neq0$, there exists $i\epsilon(1,\ldots,t)$ such that lp($g_{i})$ divides lp($f$).

A set of Gröebner bases $G=(g_{1},g_{2},..,g_{t})$ is called *reduced* if, for all *i*, the leading coefficient of $g_{i}$is 1, and no non-zero term in $g_{i}$ is divisible by any lp($g_{j}$) for any $j\neq i.$

For a fixed monomial order, every nonzero ideal $I$ has a unique reduced Gröebner basis with respect to that order*.*

*Existence*: Every non-zero ideal *I* of $k\left[ x_{1},x_{2},\ldots,x_{n} \right]$ has a Gröebner basis.

The *ideal membership* problem is defined as follows:

Let$I=<f_{1},f_{2},..,f_{s}>$ be an ideal of $k\left[ x_{1},x_{2},\ldots,x_{n} \right].$Given $f\epsilon k\left[ x_{1},x_{2},\ldots,x_{n} \right]$, determine whether *f* is in *I*.

The question is answered by the Gröebner basis:

Let *G* be a Gröebner basis for the ideal *I*. Then $f\in I$ if and only if the remainder of $f$ after division by $G$ is zero i.e. $R\left( f,G \right)=0$. Thus,$f$ can be expressed as a linear combination of the elements of the Gröebner basis:

$f=p_{1}g_{1}+\ldots+p_{t}g_{t}.$
